# Noise Perception, Sensitivity, and Patient Outcomes During Cesarean Delivery

**DOI:** 10.1155/anrp/5707084

**Published:** 2025-04-07

**Authors:** Unyime Ituk, Erik Anderson, Michelle N. Bremer Gama, Carl Skoog

**Affiliations:** ^1^Department of Anesthesia, University of Iowa, 200 Hawkins Dr, Iowa, Iowa, USA; ^2^Department of Anesthesiology, University of Minnesota, Minneapolis, Minnesota, USA; ^3^Carver College of Medicine, University of Iowa, Iowa City, Iowa, USA; ^4^Department of Surgery, Iowa Methodist Medical Center, Des Moines, Iowa, USA

**Keywords:** cesarean delivery, neuraxial anesthesia, noise perception, noise sensitivity, operating room noise, patient stress

## Abstract

**Introduction:** Noise in the operating room (OR) is a recognized hazard, known to impair communication among staff and increase stress levels. While its effects on healthcare personnel have been studied, little is known about patient perception of noise, particularly during cesarean delivery (CD) under neuraxial anesthesia, where patients are fully conscious. This study aimed to investigate if patients undergoing CD perceive OR noise as stressful and to examine the correlation between actual noise levels and patient-reported stress.

**Methods:** This prospective observational study was conducted on women undergoing CD at the University of Iowa Hospitals and Clinics. Eligible participants had sound levels recorded in the two ORs and completed three questionnaires 24 h postsurgery: the Weinstein Noise Sensitivity Questionnaire Short Form (WNSSF), a noise perception questionnaire, and a noise-related stress questionnaire. Noise levels were measured using a Spartan model 730 noise dosimeter, and the study analyzed the correlation between noise sensitivity, perception, and stress, as well as the effect of surgery urgency on noise levels.

**Results:** Out of 70 participants, 67 were included in the final analysis. The ambient noise levels in the ORs were 53.4 and 58.5 dB, respectively. The mean noise sensitivity score was 17.6 (±3.9). Noise was perceived as very soft or soft by 23% of patients, comfortable by 67.8%, and loud or unpleasant by 9.2%. A significant association was found between noise sensitivity and stress (OR 1.15 [95 CI 1.03–1.31], *p*=0.038).

**Conclusions:** The study found that a subset of patients experienced noise-related stress during CD, particularly those with higher noise sensitivity. These findings suggest the need for interventions to reduce OR noise or manage patient perceptions of noise.

## 1. Introduction

Noise is defined as unwanted sound that can cause a disturbance. It is a recognized hazard in the healthcare environment, especially in the operating room (OR). Common sources of OR noise include the anesthesia monitors and surgical equipment, and peak noise levels can be louder than standing next to a busy freeway [1]. Prior studies have showed that there is a relationship between OR noise, impaired communication among staff, and frequent occurrence of distracting events [2, 3]. OR noise is also associated with an increased level of stress in healthcare personnel when working in a noisy OR [2]. However, few studies have investigated patient perception and sensitivity to noise in the OR [4, 5]. This is probably because most surgical patients receive general anesthesia and are not conscious while in the OR, except for a brief period before induction of anesthesia. However, cesarean delivery (CD) is typically performed under neuraxial anesthesia with no concomitant administration of sedating or hypnotic drugs. These patients are therefore fully conscious and are often anxious about the outcome for their newborn and themselves. Patients are even more anxious if presenting for emergency delivery compared to a planned or scheduled CD. Therefore, OR noise may exacerbate patient discomfort and stress. A prior study reported mean noise levels during CD ranging from 61 to 72 dB, depending on the urgency of the surgery [6]. In the general surgical population, it is reported that over 50% of patients experienced noise in the OR and 16% of them were distressed by the noise [5]. It is likely that noise-related stress is experienced by patients during CD.

The aim of this study was to determine whether patients undergoing CD under neuraxial anesthesia perceive the noise in the OR as stressful, and whether there is a correlation between actual noise levels and their perception of it. We hypothesize that patients with higher noise sensitivity will report greater noise perception and increased stress related to noise.

## 2. Methods

This prospective observational study was conducted at the University of Iowa Hospitals and Clinics in patients undergoing CD. The study was approved by the University of Iowa Institutional Review Board (IRB #202012134). This study adheres to the Strengthening the Reporting of Observational studies in Epidemiology (STROBE) guidelines (Appendix strobe checklist table legend). Patients were screened for eligibility prior to entering the OR for delivery. The inclusion criteria included pregnant women aged ≥ 18 years, singleton term (≥ 37 weeks), having a CD under neuraxial anesthesia, and fluent in English. The exclusion criteria included the American Society of Anesthesiologists (ASA) physical Status 4, patients who receive amnestic drugs (e.g., midazolam, dexmedetomidine, propofol, or nitrous oxide), failed spinal or epidural anesthesia requiring conversion to general anesthesia, history of psychiatric illness that may influence responses to the questionnaire, history of hearing impairments or use of hearing aids, and history of chronic pain or pain syndromes. The ambient sound level in the Labor and Delivery unit ORs was measured prior to subject enrollment.

Eligible subjects had sound levels measured using a noise dosimeter on entry to the OR and during the CD. The subjects and members of the healthcare team in the OR were not informed that noise levels in the OR were being recorded to avoid behavior changes (Hawthorne effect). Sound was measured using a Spartan model 730 noise dosimeter (Larson Davis, Depew NY, USA). Specifically, two metrics were recorded: the LAeq, which represents the average noise level over a specified period using *A*-weighting and adjusts the measurements to reflect the sensitivity of the human ear to different frequencies; LCpeak, which measures the highest instantaneous noise level during a measurement period. It uses *C*-weighting, which provides a flatter frequency response compared to *A*-weighting. The dosimeter was calibrated before each use, and its microphone was attached to the midpoint of the head section of the operating table. During sound recording, there was continuous visual observation of the microphone by research personnel to prevent any material that may cause sound artifacts when in contact with microphone or cause a disconnection from the dosimeter. Sound levels were recorded from time of patient entry to the OR until the end of surgery. The time in the OR was divided into four phases: from patient entry into the OR to positioning for neuraxial anesthesia (*T*0), from neuraxial block placement to skin incision (*T*1), from skin incision to delivery of the neonate (*T*2), and from delivery of the neonate to skin closure (*T*3). All patients in the study received our institution's standard spinal anesthesia dose which is 12 mg of 0.75% hyperbaric bupivacaine, 15 mcg of fentanyl, and 150 mcg of preservative-free morphine. They also had a variable weight-based prophylactic phenylephrine infusion for the management of spinal hypotension and dual-agent antiemetics.

Patients were approached 24 h after their CD for consent to participate in the study. Those that agreed and signed the consent form were given three questionnaires: the Weinstein Noise Sensitivity Questionnaire Short Form (WNSSF), a noise perception questionnaire, and a noise-related stress questionnaire. The WNSSF assesses noise sensitivity based on patient responses to five statements about noise sensitivity, rated on a six-point Likert scale from 1 (*strongly disagree*) to 6 (*strongly agree*) [7]. The noise perception questionnaire asked patients to rate the noise they perceived in the OR and to recall which phase of their time in OR had the loudest noise. Noise perception was rated on a seven-point scale, from one (*inaudible*) to 7 (*unpleasant*). Lastly, patients were administered the noise-related stress questionnaire, consisting of one question assessing the patient's overall stress level related to sound in the OR. The noise-related stress questionnaire consisted of one question about the patient's overall stress level due to OR noise, rated on a five-point Likert scale from 1 (*not at all stressful*) to five (*intolerably stressful*). All questionnaires are listed in the Supporting Information section. The questions on the noise perception and noise-related stress questionnaires were assessed to determine if they cover all relevant aspects of the outcome being measured and to ensure there were appropriate and understandable for the target population. A pilot sample involving 10 women was carried out to evaluate the feasibility and applicability of the questionnaires and refine items based on responses and feedback. The main outcome of interest was the correlation between noise sensitivity, noise perception, and patient stress.

### 2.1. Statistical Analysis

Summary statistics were calculated for all study variables. Categorical measures were reported as counts and percentages, while continuous variables were summarized as means with standard deviations or medians with interquartile ranges. Associations between categorical responses were analyzed using contingency tables and Fisher's exact test. The effects of individual and combined sensitivity measures on patient stress were modeled using cumulative logistic regression. Odds ratios with 95% confidence intervals were calculated for each predictor, along with *p* values. Similar models were used to analyze the relationship between peak OR noise and patient stress. The Wilcoxon rank-sum test was used to assess whether the urgency of the surgery was related to peak noise levels. All testing was conducted at the alpha = 0.05 significance level. We estimated that 60% of patients who perceived the OR as noisy would find it stressful, compared to 20% of patients who did not, with a sample size ratio of 30/70 (0.429). A sample size of 56 patients (26 in the “experience noise” group and 30 in the “nonexperience noise” group) was calculated to achieve 90% power for a one-tailed test with alpha = 0.05. To account for protocol violations, we planned to enroll 70 patients in total.

## 3. Results

The study was conducted between June 2021 and April 2023, with 127 women assessed for eligibility. A total of 65 participants completed the study after excluding those who did not meet the criteria or had protocol violations. The demographics and characteristics of those included are summarized in Table 1. The study took place in two ORs, where ambient noise levels were measured as 53.4 dB in OR 1 and 58.5 dB in OR 2. These measurements were taken over 10 min when the ORs were unoccupied with all equipment (including anesthesia machine, suction device, monitors, fluid warmers, and drug refrigerator) in standby mode and the air exchange system on. The LAeq noise levels in the OR from time of patient entry ranged from 60.2 to 68.4 dB, while the LCpeak noise pressure varied between 79.1 and 122.9 dB.

The mean noise sensitivity score on the WNSSF was 17.6 (±3.9). Noise was described as “very soft” or “soft” by 23% of patients, “comfortable” by 67.8%, and “loud or unpleasant” by 9.2%.  Table 2 shows the summary of the frequency weighted continuous sound level (LAeq) and peak sound pressure (LCpeak) in the OR stratified by patient perceived sound intensity. The highest noise levels were reported after the delivery of the baby by 41.5% of patients, while 16.9% noted loud noises before delivery, 13.9% during neuraxial anesthesia placement, 3% just before they left the OR, and 24.6% could not remember. There was no significant association between the phase of care and the reported time of loudest noise (*p*=0.17).

A significant association was found between perceived noise intensity and stress levels (*p*=0.004), this is summarized in Table 3. Logistic regression indicated that each unit increase in noise sensitivity was associated with a 15% increase in the odds of experiencing noise-related stress (OR 1.15, 95% CI: 1.03–1.31, *p*=0.038). However, the small number of emergency CDs (*n* = 2) limited the analysis of noise differences based on urgency.

## 4. Discussion

The findings of our study indicate that while most patients perceived the sound levels in the OR as comfortable, 9.2% of them found the noise levels to be loud or unpleasant. Notably, there was a clear association between the perceived noise and noise-related stress, particularly for patients with higher noise sensitivity scores. This supports our hypothesis that patients with increased noise sensitivity are more likely to report higher levels of noise perception and stress. The association between noise sensitivity and stress is critical, as it underscores the impact of subjective noise perception, rather than just the objective noise levels, on patient comfort. For example, a patient with a noise sensitivity score of 25/30 versus one with a score of 20/30 would have more than double the odds (OR 2.01) of experiencing noise-related stress. This emphasizes the need to address environmental factors such as noise in ORs, particularly for conscious patients undergoing procedures such as CD under neuraxial anesthesia.

Previous studies have highlighted the detrimental effects of noise in the OR on both healthcare personnel and patients. Katz noted that noise levels in the OR often exceed those of a busy highway, contributing to stress and impaired communication among staff [1]. Similarly, McMullan et al. linked OR noise and distractions with extended procedure times and impaired team performance [8]. In our study, the LCpeak noise levels in the OR reached up to 122 dB which is comparable to the noise from a gas-powered chainsaw [9]. A recent survey study seeking to identify the severity and contributing factors for noise in the OR reported that noise occurs at unacceptable levels across all the institutions who responded to the survey [10]. The authors also reported that only 20% of institutions implemented noise mitigation strategies and only 13% of respondents found these measures effective in reducing OR noise. A holistic approach to noise mitigation, including implementing noise control policies, incorporating noise-reducing materials in OR design, and educating staff about the impacts of noise, has been suggested to enhance effectiveness [11].

Patients who described the noise as “very soft” or “soft” generally did not find it stressful. However, as the noise was perceived as “comfortable” or louder, an increasing number of patients reported feeling slightly stressed or stressed. This suggests that even when actual sound levels remain within a certain range, individual perceptions of loudness and comfort may vary, influencing stress responses. These findings are consistent with those reported by Hasfeldt et al., who found that 10% of general surgical patients perceived preanesthesia noise as disruptive and stressful [4]. This observation further reinforces the importance of patient-centered approaches to manage the OR environment, particularly for awake patients such as those undergoing CD, where anxiety levels may already be heightened due to concerns about both maternal and fetal outcomes.

While our study provides valuable insights, several limitations should be considered when interpreting the results. First, the study was conducted at a single institution, which may limit the generalizability of the findings to other settings. Differences in OR design, equipment, and staff behavior across institutions could result in different noise levels and patient perceptions. Furthermore, the study cohort consisted of women undergoing CD, who may experience unique stressors related to childbirth, potentially affecting their sensitivity to noise.

Another limitation is the potential for recall bias, as patients were asked to retrospectively report their noise perception and stress levels 24 h postsurgery. Factors such as pain, anesthesia recovery, or delivery outcomes could have influenced their recollection of intraoperative noise. Additionally, the study did not account for baseline anxiety or psychological factors, which may also affect noise sensitivity and stress.

Despite these limitations, the results provide valuable insights into the impact of noise on patient stress during surgery, particularly in conscious patients. Interventions aimed at reducing OR noise or managing patient perceptions of noise could improve the patient experience. Strategies such as optimizing OR design, reducing unnecessary noise, or providing noise-canceling devices may help mitigate noise-related stress. Moreover, our study suggests the potential for tailoring care based on individual noise sensitivity. Identifying patients with higher noise sensitivity could allow for personalized interventions to reduce stress and improve outcomes. Future research should investigate the efficacy of such interventions and explore the broader impact of noise management on patient satisfaction and surgical outcomes.

## 5. Conclusion

Our study highlights the importance of considering environmental factors such as noise in the OR, especially for patients undergoing procedures under neuraxial anesthesia. Addressing these factors may significantly improve the surgical experience for patients, reducing anxiety and stress, and potentially enhancing overall patient care.

## Figures and Tables

**Table 1:** Demographic and obstetric data of the study cohort (*n* = 65).

Age (years)	31.4 ± 5.5
Body mass index (kg/m^2^)	59 ± 11.6
Race	
Caucasian	59 (90.8)
African American/black	3 (4.6)
American Indian/Alaskan native	2 (3.1)
Asian	1 (1.5)
Ethnicity	
Hispanic	4 (6.2)
Non-Hispanic	61 (93.8)
Gestational age (weeks)	38.8 (38.1–39.3)
Nulliparous	12 (18.5)
Indications for CD	
Breech	14 (21.5)
Previous CD	38 (58.5)
Failure to progress in labor	4 (6.2)
NRFHT	3 (4.6)
Others	6 (9.2)

*Note:* Values are expressed as mean ± SD, median (IQR), and number (%) as appropriate.

Abbreviations: CD, cesarean delivery; NRFHT, nonreassuring fetal heart trace.

**Table 2:** Measured weighted continuous sound level and peak sound pressure stratified by patient's perceived noise intensity.

Perceived noise intensity	*n*	LAeq (dB)	LCpeak (dB)
Very soft	8	64.3 (61.5–65.7)	83 (80.3–110.8)
Soft	7	62.6 (62.3–65.4)	83.2 (82.1–104.7)
Comfortable	44	63.5 (62.4–64.7)	93 (82.9–111.3)
Loud	5	63.9 (62.6–64.2)	109.8 (84.9–110.3)
Unpleasant	1	62.8 (61.1–64.4)	81.6 (80.0–82.1)

*Note:* values are median (IQR).

**Table 3:** Patient-reported noise perception compared to reported noise-related stress.

Perceived noise intensity	Not at all stressful	Slightly stressful	Stressful	Total
Very soft	8	0	0	8
Soft	5	2	0	7
Comfortable	29	12	3	44
Loud	0	4	1	5
Unpleasant	0	0	1	1
	42	18	5	65

*Note:* Fisher's exact test result *p*=0.0040.

## Data Availability

The data that support the findings of this study are available on request from the corresponding author. The data are not publicly available due to privacy or ethical restrictions.

## Supporting Information

The Weinstein noise sensitivity short form, noise perception questionnaire, and noise-related stress questionnaire are included.
