# Supplementary material for: Noise Perception, Sensitivity, and Patient Outcomes During Cesarean Delivery
Source: Anesthesiol Res Pract. 2025 Apr 7;2025:5707084. doi: 10.1155/anrp/5707084 (PMC11996264; doi:10.1155/anrp/5707084)
Supplement: Supporting Information — Additional supporting information can be found online in the Supporting Information section. [file 5707084.f1.zip › Noise perception Questionnaire.docx]

**Noise Perception Questionnaire**

*Instructions: Please answer the following questions that best describe your experience in the labor and delivery operating room.*

1. During your time in the operating room, from when you entered until your baby was born which of the following best describes the sounds you heard on a scale of 1 – 7? (below are the descriptors for each number on the scale)
2. Inaudible
3. Very soft
4. Soft
5. Comfortable
6. Loud
7. Very loud
8. Unpleasant
9. At which time during your delivery did you hear the loudest sound
   1. While getting your anesthesia
   2. Before delivery of your baby
   3. After delivery of your baby
   4. Just before you left the operating room
   5. Can’t remember
