# Supplementary material for: Noise Perception, Sensitivity, and Patient Outcomes During Cesarean Delivery
Source: Anesthesiol Res Pract. 2025 Apr 7;2025:5707084. doi: 10.1155/anrp/5707084 (PMC11996264; doi:10.1155/anrp/5707084)
Supplement: Supporting Information — Additional supporting information can be found online in the Supporting Information section. [file 5707084.f1.zip › Noise Related Stress in the Operating Room.docx]

**Noise Related Stress in the Operating Room**

Regarding your experience in the operating room during the delivery of your baby please answer the following question.

From the time you entered the operating room until your baby was born, did you find the sound in operating room stressful?

Please choose one option below

- 1. Not at all stressful
  2. Slightly stressful
  3. Stressful
  4. Extremely stressful
  5. Intolerably stressful
