# Supplementary material for: Noise Perception, Sensitivity, and Patient Outcomes During Cesarean Delivery
Source: Anesthesiol Res Pract. 2025 Apr 7;2025:5707084. doi: 10.1155/anrp/5707084 (PMC11996264; doi:10.1155/anrp/5707084)
Supplement: Supporting Information — Additional supporting information can be found online in the Supporting Information section. [file 5707084.f1.zip › Weinstein Noise Sensitivity Short Form.docx]

**Weinstein Noise Sensitivity Short Form**

*For the following 5 questions, which answer best describes your sensitivity to noise.*

1. I am sensitive to noise.

| Strongly Disagree | Disagree | Slightly Disagree | Slightly Agree | Agree | Strongly Agree |
| --- | --- | --- | --- | --- | --- |

1. I find it hard to relax in a place that is noisy.

| Strongly Disagree | Disagree | Slightly Disagree | Slightly Agree | Agree | Strongly Agree |
| --- | --- | --- | --- | --- | --- |

1. I get mad a people who make noise that keeps me from falling asleep or getting work done.

| Strongly Disagree | Disagree | Slightly Disagree | Slightly Agree | Agree | Strongly Agree |
| --- | --- | --- | --- | --- | --- |

1. I get annoyed when my neighbors are noisy.

| Strongly Disagree | Disagree | Slightly Disagree | Slightly Agree | Agree | Strongly Agree |
| --- | --- | --- | --- | --- | --- |

1. I get used to most noises without much difficulty.

| Strongly Disagree | Disagree | Slightly Disagree | Slightly Agree | Agree | Strongly Agree |
| --- | --- | --- | --- | --- | --- |
